# Novel Soil-Derived Beta-Lactam, Chloramphenicol, Fosfomycin and Trimethoprim Resistance Genes Revealed by Functional Metagenomics

**DOI:** 10.3390/antibiotics10040378

**Published:** 2021-04-03

**Authors:** Inka Marie Willms, Maja Grote, Melissa Kocatürk, Lukas Singhoff, Alina Andrea Kraft, Simon Henning Bolz, Heiko Nacke

**Affiliations:** Department of Genomic and Applied Microbiology and Göttingen Genomics Laboratory, Institute of Microbiology and Genetics, Georg-August University of Göttingen, D-37077 Göttingen, Germany; inka-marie.willms@basf.de (I.M.W.); maja.grote@stud.uni-goettingen.de (M.G.); melissa.kocatuerk@stud.uni-goettingen.de (M.K.); l.singhoff@stud.uni-goettingen.de (L.S.); alinaandrea.kraft@stud.uni-goettingen.de (A.A.K.); simonhenning.bolz@stud.uni-goettingen.de (S.H.B.)

**Keywords:** antibiotic resistance genes, soil, functional metagenomics, beta-lactam resistance, chloramphenicol resistance, fosfomycin resistance, trimethoprim resistance

## Abstract

Antibiotic resistance genes (ARGs) in soil are considered to represent one of the largest environmental resistomes on our planet. As these genes can potentially be disseminated among microorganisms via horizontal gene transfer (HGT) and in some cases are acquired by clinical pathogens, knowledge about their diversity, mobility and encoded resistance spectra gained increasing public attention. This knowledge offers opportunities with respect to improved risk prediction and development of strategies to tackle antibiotic resistance, and might help to direct the design of novel antibiotics, before further resistances reach hospital settings or the animal sector. Here, metagenomic libraries, which comprise genes of cultivated microorganisms, but, importantly, also those carried by the uncultured microbial majority, were screened for novel ARGs from forest and grassland soils. We detected three new beta-lactam, a so far unknown chloramphenicol, a novel fosfomycin, as well as three previously undiscovered trimethoprim resistance genes. These ARGs were derived from phylogenetically diverse soil bacteria and predicted to encode antibiotic inactivation, antibiotic efflux, or alternative variants of target enzymes. Moreover, deduced gene products show a minimum identity of ~21% to reference database entries and confer high-level resistance. This highlights the vast potential of functional metagenomics for the discovery of novel ARGs from soil ecosystems.

## 1. Introduction

Antibiotic resistance has emerged as one of the most serious threats to public health since multidrug-resistant pathogens cause severe problems in hospitals and animal husbandry worldwide. This led to a stronger focus on non-clinical resistomes, including soil ecosystems, which are considered to represent a major source of antibiotic resistance genes (ARGs). So far, many of the various ARGs, harbored by the enormous diversity of soil microorganisms, still remain undiscovered. Functional metagenomics is considered as a very promising approach to improve this situation, as it allows the identification of entirely novel as well as unknown variants of target genes [1,2,3].

Within a previous study focusing on soil resistomes [4], more than thirty novel antibiotic resistance determinants were discovered by applying function-based metagenomic library screening, demonstrating that non-clinical ARG reservoirs are far from being extensively explored. The discovered resistance determinants include a potential dihydropteroate synthase (FolP^SMZ B27^), which confers resistance to sulfonamides and shared the greatest homology with reference database entries representing uncultured bacteria affiliated to the candidate phyla radiation group (CPR) [4]. Besides access to ARGs carried by the uncultured microbial majority, functional metagenomics also allowed the identification of genes previously not associated with antibiotic resistance. For instance, Allen et al. [5] reported that introduction of a soil metagenome-derived response regulator gene into *Escherichia coli* led to an antibiotic resistance phenotype by decreasing the sensitivity toward carbenicillin, a beta-lactam antibiotic.

Beta-lactam antibiotics represent one of the most widely used antibiotic classes [6,7]. These antibiotics are known to inhibit cell wall formation and resistance can occur through multiple molecular mechanisms, including the synthesis of efflux pumps, alteration of the molecular target and production of beta-lactamases [8,9]. For instance, the clinically relevant extended spectrum beta-lactamase coding gene *shv* has been detected in soil [10]. Apart from beta-lactam antibiotics, fosfomycin, which represents the so far only available epoxide antibiotic, also inhibits cell wall formation [11,12]. Notably, fosfomycin belongs to the so-called last resort antibiotics, which often represent the only treatment option when other drugs fail to efficiently fight infections [13]. Resistance toward this antimicrobial compound can arise via amino acid substitutions within the active site of the target enzyme UDP-N-acetylglucosamine enolpyruvyl transferase (MurA), reduced uptake, or production of fosfomycin-modifying enzymes [11,14]. So far, fosfomycin resistance was rarely considered in surveys focusing on soil resistomes. Nevertheless, genes encoding fosfomycin-modifying enzymes such as *fosK* [15] have already been detected in soil and a fosfomycin-resistant MurA variant has been identified based on soil metagenomic library screening [16].

In this study, both, beta-lactam as well as fosfomycin resistances were targeted during function-based screening of soil metagenomic libraries. Moreover, we aimed to identify resistances against chloramphenicol, a potent inhibitor of bacterial protein biosynthesis, and trimethoprim, a synthetic antimicrobial agent functioning as antifolate. With respect to chloramphenicol, antibiotic-modifying enzymes, e.g., different types of chloramphenicol acetyltransferases, can cause resistance [17,18]. Genes encoding such enzymes have been detected in, e.g., pristine Antarctic soil resistomes [19]. Additionally, ribosome protection as well as efflux via specific or multidrug transporters has been reported [17,20]. With respect to soil bacteria, efflux-mediated chloramphenicol resistance has, for instance, been identified in *Streptomyces coelicolor* [21]. In case of trimethoprim, resistance is commonly caused by drug-resistant variants of the target enzyme dihydrofolate reductase (DHFR) [22,23]. Both, drug-resistant type I as well as type II DHFRs have already been discovered in soil [24,25].

Here, we report the identification of three novel beta-lactam, a previously undiscovered chloramphenicol, a new fosfomycin, as well as three so far unknown trimethoprim resistance genes. These genes were derived from forest and grassland soil metagenomic libraries and are affiliated to a variety of bacterial phyla.

## 2. Results and Discussion

### 2.1. Screening of Soil Metagenomic Libraries Enabled the Discovery of Eight Novel ARGs

To identify novel genes encoding beta-lactam (ampicillin and ceftazidime), chloramphenicol, fosfomycin and trimethoprim antibiotic resistance, soil-derived metagenomic libraries, containing 54,320 to 559,000 clones (Table 1), were subjected to function-based screening. The quality of these libraries was controlled by determining the average insert sizes and the percentage of insert-bearing *Escherichia coli* clones. Average insert sizes of soil DNA-containing plasmids ranged from 3.4 to 6.7 kb, and the frequency of clones carrying such inserts was at least 80% (Table 1).

A total of eight positive *E. coli* clones, harboring plasmids listed in Table 1, were recovered by applying function-based screening. Subsequently, each insert with respect to these plasmids was sequenced and taxonomically classified, which revealed in all cases a bacterial origin (Table S1). For instance, insert sequences affiliated with *Acidobacteria*, *Bacteroidetes* and *Proteobacteria* were identified (Table S1). This demonstrates that the metagenomic library host *E. coli* allows identification of ARGs carried by phylogenetically divergent soil bacteria. Even an insert affiliated with *Actinobacteria*, which typically show a high-GC content and predicted transcriptional incompatibilities with *E. coli*, was detected (Table S1). Nevertheless, it cannot be excluded that some of the ARGs, present in our metagenomic libraries, are not expressed by the host *E. coli* due to, e.g., codon bias [1,28].

Subcloning of candidate genes (Table 2), encoded by plasmids of positive clones, and subsequent susceptibility testing confirmed that these genes confer antibiotic resistance to *E. coli*. The deduced gene products comprised 165 to 443 amino acids, and sequence identities to the closest database entries ranged from approximately 21% to 92% over the full length proteins (Table 2). For one (SEG8_Cef01) of the eight deduced gene products, a signal peptide was predicted.

### 2.2. Trimethoprim Resistance Mediated by Soil-Derived DHFRs and a Pteridine Reductase

The plasmids pCR4_SEG8_tri01, pCR4_SEG8_tri02 and pCR4_AEW5_tri01 confer resistance to the synthetic antibiotic trimethoprim (Table 3). Trimethoprim represents an antifolate, which selectively affects bacterial cells [22]. This antibiotic fits well in the nucleotide-binding site of different bacterial DHFRs such as the DHFR harbored by *E. coli* [29]. Drug-resistant variants of these enzymes are a commonly occurring bacterial feature to overcome bacteriostatic effects of trimethoprim [30]. We identified two gene products, SEG8_Tri01 and SEG8_Tri02, showing similarity to a DHFR from *Flavobacterium terrigena* and a *Flammeovirgaceae* bacterium, respectively (Table 2). To our knowledge, it has so far not been tested if these two DHFRs confer trimethoprim resistance.

**Figure 1:**
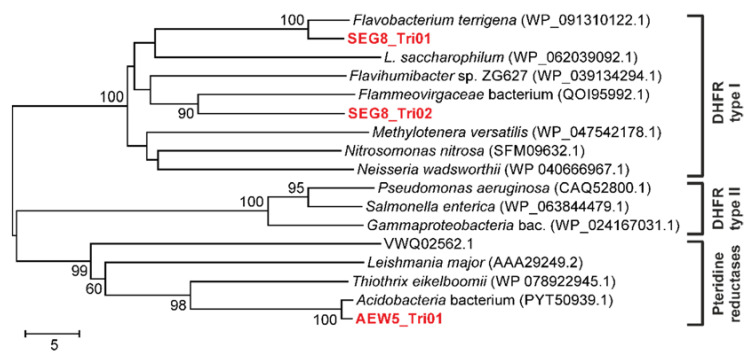
Neighbor-joining phylogenetic tree based on amino acid sequences of AEW5_Tri01, SEG8_Tri01, SEG8_Tri02, and different type I as well as type II dihydrofolate reductases (DHFRs) and pteridine reductases. Bootstrap values ≥50, based on 1000 iterations, are shown at branching points. Branches are annotated with the identified taxon names and accession numbers of the different proteins are given in parentheses. Abbreviations: *L. saccharophilum*, *Lentimicrobium saccharophilum*; bac., bacterium.

Type I and type II DHFRs, exhibiting a length of ~180 and ~80 amino acids, respectively, have been reported [24,31,32]. Phylogenetic analysis (Figure 1) revealed that both, SEG8_Tri01 (165 amino acids) and SEG8_Tri02 (166 amino acids), represent type I DHFRs. Importantly, we also identified a gene product (AEW5_Tri01), conferring trimethoprim resistance, which clusters with none of the two DHFR types (Figure 1).

This gene product shows similarity to a pteridine reductase harbored by an *Acidobacteria* representative (Table 2). It has so far not been reported if this pteridine reductase can confer trimethoprim resistance, but it is known that some pteridine reductases can contribute to antifolate drug resistance by providing a molecular bypass of DHFR inhibition [33]. Therefore, for an effective therapy, DHFRs as well as pteridine reductases carried by target pathogens have to be inhibited. To the best of our knowledge, we report here for the first time a trimethoprim-resistant and soil-derived bacterial pteridine reductase identified by function-based metagenomic library screening. In previous studies, metagenomic library screens based on trimethoprim as selective component led to the discovery of numerous type I DHFRs and also thymidylate synthase as well as DHFR type II representatives [24,31,34].

### 2.3. Identification of a Novel Subclass B1 Metallo-Beta-Lactamase

Function-based screening of metagenomic library SEG8 led to the identification of plasmid pLSEG8_cef01, which confers resistance to ceftazidime, a third generation cephalosporin. Subcloning of *SEG8_cef01* revealed that this gene encodes ceftazidime resistance along with decreased sensitivity to cephalexin and cefoxitin, representing cephalosporins of the first and second generation, respectively (Table 3). The gene product SEG8_Cef01 showed only ~62% identity to a beta-lactamase from an uncultured bacterium (Table 2).

**Figure 2:**
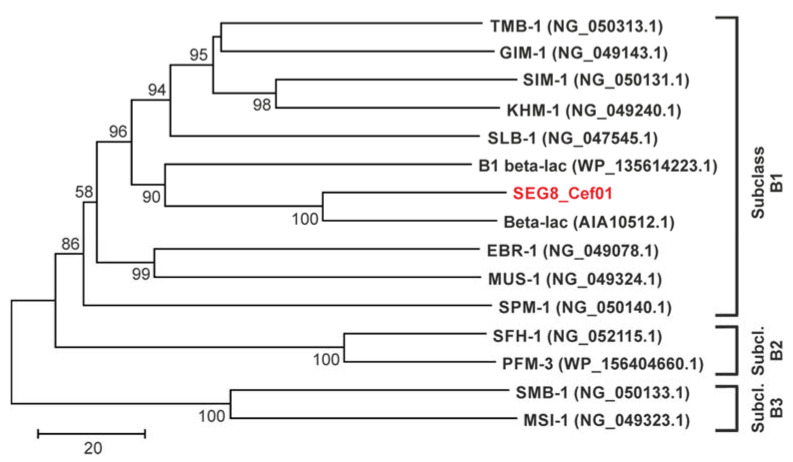
Neighbor-joining phylogenetic tree based on amino acid sequences of SEG8_Cef01 and other metallo-beta-lactamases. Besides SEG8_Cef01, its closest related reference database entry, and different representatives of subclasses B1, B2 and B3 were considered. Bootstrap values ≥50, based on 1000 iterations, are shown at branching points. Accession numbers of the different beta-lactamases are given in parentheses. Abbreviations: Subcl., Subclass; Beta-lac, Beta-lactamase.

In order to further characterize SEG8_Cef01, we performed a phylogenetic analysis, which revealed that it belongs to the metallo-beta-lactamase (MBL) subclass B1 (Figure 2).

**Figure 3:**
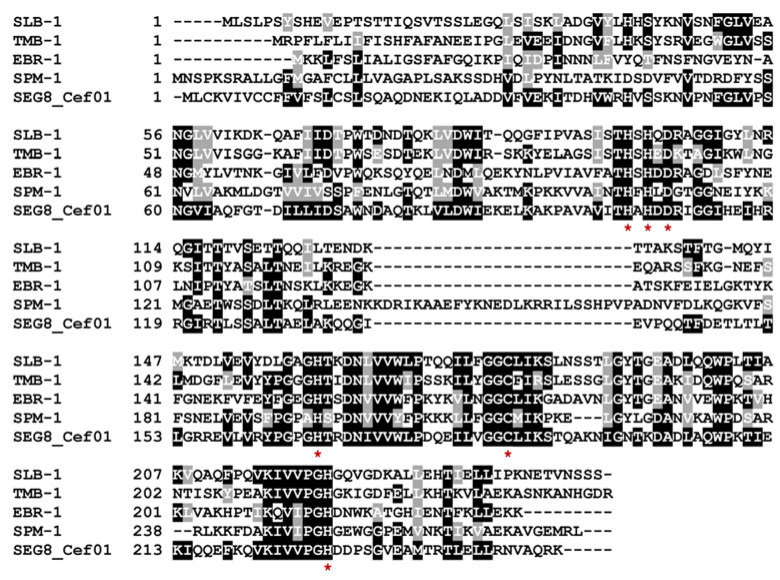
Amino acid alignment of SEG8_Cef01 and known subclass B1 metallo-beta-lactamases. Zinc-binding amino acids are indicated by red asterisks below the specific positions. Accession numbers of the different beta-lactamases are provided in Figure 2.

All MBLs catalyze the hydrolysis of beta-lactams via a non-covalent mechanism in which one or two equivalents of bound zinc ions promote formation of a nucleophilic hydroxide [35]. The three different MBL subclasses (B1, B2 and B3) are primarily distinguished by differences in the primary zinc coordination shell [36]. An alignment including SEG8_Cef01 and other subclass B1 MBLs showed that the here identified enzyme contained all zinc-binding amino acids typical for subclass B1 (Figure 3).

Among the three different MBL subclasses, subclass B1 emerged as the most clinically significant [35,37]. It includes VIMs (Verona integron-encoded MBLs), IMPs (imipenemases) and NDMs (New Delhi MBLs) [35]. In contrast to serine beta-lactamases, MBLs use a non-covalent catalytic mechanism, mentioned above, and therefore are not susceptible to serine beta-lactamase-inhibiting drugs such as sulbactam, tazobactam, clavulanic acid and avibactam [35].

The here detected subclass B1 MBL shows that mining environmental resistomes by applying functional metagenomics represents a valuable approach to trace antibiotic resistance determinants with potential clinical relevance. Notably, a signal peptide has been predicted with respect to SEG8_Cef01. The signal peptide is potentially involved in the translocation of SEG8_Cef01 across the inner membrane, enabling inactivation of beta-lactams in the periplasmic space. This theory fits to the fact that beta-lactamases are typically located in the periplasm of bacteria [38].

### 2.4. Efflux-Mediated Chloramphenicol Resistance Encoded by SEW5_chl01

Efflux via specific or multidrug transporters can potentially lead to chloramphenicol resistance [17,20]. The chloramphenicol resistance protein, SEW5_Chl01, identified in this study is most closely related to a Bcr/CflA subfamily drug resistance transporter derived from an uncultured bacterium (Table 2). Drug resistance transporter Bcr/CflA subfamily proteins belong to the major facilitator superfamily (MFS), which comprises representatives conferring resistance to multiple antibiotics [39,40].

MFS efflux proteins are widely distributed among both, Gram-positive as well as Gram-negative bacteria [41,42], and in some cases, these proteins have been reported to decrease susceptibility toward rifampicin [24,39]. With respect to *E. coli* harboring SEW5_Chl01, no resistance toward rifampicin was detected (Table 3). Nevertheless, besides resistance to chloramphenicol, SEW5_Chl01 conferred slightly decreased sensitivity toward tetracycline (Table 3).

It is known that efflux pump determinants are in some cases carried by mobile genetic elements [40,43,44]. Here, we found that *SEW5_chl01* is flanked by a gene encoding a deduced protein with similarity to a transposase (Supplementary Table S2). Thus, it is possible, that *SEW5_chl01* efficiently spreads within soil bacterial communities via horizontal gene transfer (HGT).

### 2.5. AEW1_Fos01 Confers Resistance to the Last Resort Antibiotic Fosfomycin

Fosfomycin, the only so far available epoxide antibiotic, has been categorized as critically important by the WHO and is used to treat complicated infections caused by different multidrug-resistant pathogens such as *Haemophilus influenzae* and *Streptococcus pyogenes* strains [11,13,45]. Here, we detected a gene product, designated AEW1_Fos01, conferring resistance to this broad spectrum antibiotic and showing approximately 92% identity to a MurA variant from a *Mucilaginibacter* representative (Table 2 and Table 3).

**Figure 4:**
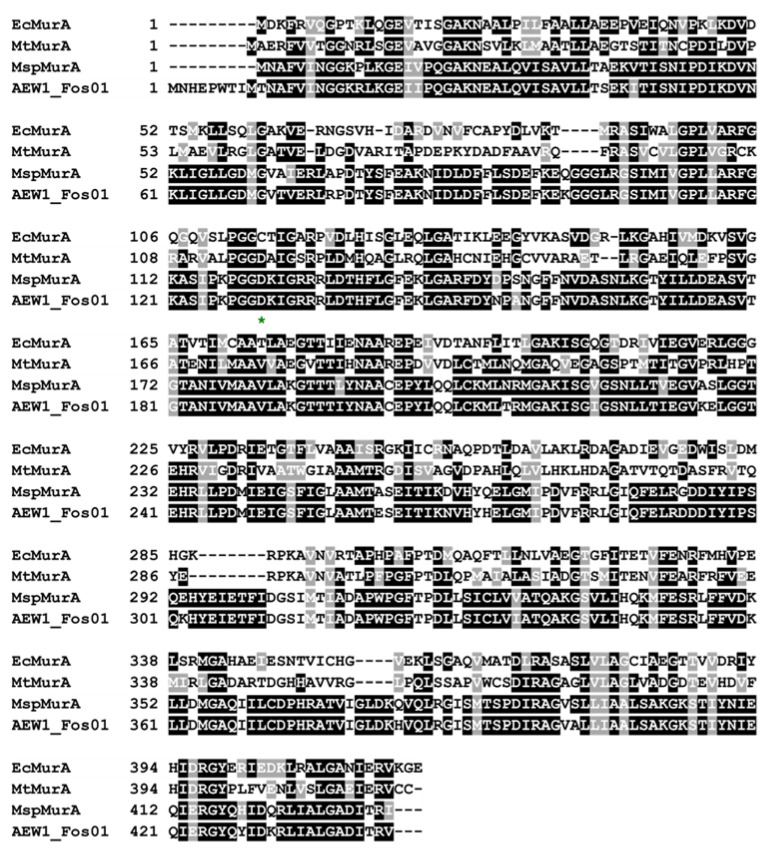
Amino acid alignment of AEW1_Fos01 and known MurA representatives. The active site Cys/Asp residue is indicated by a green star below the specific position. Besides AEW1_Fos01, the alignment includes *Escherichia coli* MurA (EcMurA, accession number: AAC76221), *Mycobacterium tuberculosis* H37Rv MurA (MtMurA, accession number: CAA65472) and a *Mucilaginibacter* sp. MurA (MspMurA, accession number: HAL80920).

MurAs catalyze the first step with respect to bacterial peptidoglycan synthesis and are in some cases inhibited by fosfomycin. Nevertheless, a single amino acid substitution within the active site of MurAs can lead to resistance toward this antibiotic, which is used to treat urinary tract and bladder infections [16,45,46]. Based on a sequence alignment including AEW1_Fos01 and different MurAs, we identified this amino acid substitution in the here detected fosfomycin resistance-conferring enzyme (Figure 4). More precisely, aspartic acid instead of cysteine was located at a specific position in the active site (see Figure 4). Noteworthy, the insert harboring *AEW1_fos01* encodes a protein with similarity to a transposase (Table S2), which might enable HGT. This illustrates the potential of ARGs present in soil ecosystems to become clinically relevant in the future. Especially, mobile genes conferring resistance to last resort antibiotics are problematic as they can foster the rise of pathogens, which cannot be efficiently treated with available antibiotics.

### 2.6. AEW4_Amp01 and SEG8_Amp01 Evoke Resistance toward Different Penicillins

Among the gene products conferring antibiotic resistance detected in this study, AEW4_Amp01 and SEG8_Amp01 showed the lowest identity toward reference database entries (approximately 21 and 48%, respectively). A bifunctional methionine sulfoxide reductase was the closest similar protein with respect to SEG8_Amp01 (Table 2). Furthermore, phylogenetic analysis clearly demonstrates that SEG8_Amp01 clusters with methionine sulfoxide reductase proteins (Figure S1). It is known that cell wall-active antibiotics can cause elevated synthesis of methionine sulfoxide reductases [47]. Furthermore, some of these proteins have been reported to function as efflux pumps conferring antibiotic resistance [48].

In contrast to the other detected ARGs, *AEW4_amp01* and *SEG8_amp01* were subcloned into vector pHSG398, as the vector pCR4-TOPO itself encodes ampicillin resistance. The plasmid pHSG398_SEG8_amp01 mediated no resistance to cephalosporins of the first, second and third generation (Table 4). Nevertheless, besides ampicillin, it conferred resistance to further penicillins, more precisely, carbenicillin and piperacillin (Table 4). In this context, high minimum inhibitory concentrations (MICs) were determined. More data on MICs with respect to other methionine sulfoxide reductase proteins are required to determine if these proteins can lead to MIC values, which are comparable to those determined in this study. Similar to SEG8_Amp01, AEW4_Amp01 also mediated resistance to different penicillins, but not to the considered cephalosporins (Table 4). This gene product shows very low similarity (approximately 21%) to an efflux RND (resistance nodulation division) transporter periplasmic adaptor subunit (Table 2). It is known that RND proteins are involved in efflux-mediated antibiotic resistance [49,50]. They typically comprise large periplasmic domains and form tripartite complexes with outer membrane channels and periplasmic adaptor proteins [49,51]. Here, a single gene product, AEW4_Amp01, evoked a high-level penicillin resistance. Therefore, it seems to not build a complex with other proteins as described for many RND efflux systems. Apart from efflux RND transporter periplasmic adaptor subunits, AEW4_Amp01 showed slight similarity to two beta-lactamases (the BLAST results are shown in Table S3). Nevertheless, based on phylogenetic analysis, no clustering of AEW4_Amp01 and representatives of class A, subclass B1, subclass B2, subclass B3, class C or class D beta-lactamases was observed. In this context, e.g., AmpC- and CARB-type beta-lactamases were considered. Due to the very low similarity of AEW4_Amp01 to reference database entries, the determined high MIC values with respect to penicillins cannot be adequately compared with MIC values reported in the literature. A comparison will be possible, when antibiotic resistance proteins with a higher similarity to AEW4_Amp01 are identified and comprehensively analyzed. Considering the high diversity of soil resistomes, it can be assumed that such resistance proteins can be discovered in these resistomes in future surveys.

The insert of pLAEW4_amp01 encodes a protein with similarity to a homing endonuclease (Table S2). Homing endonucleases are small proteins which often occur in mobile genetic elements and contribute to HGT [52]. Therefore, it is possible that *AEW4_amp01* is not only harbored by the host of the here detected environmental DNA fragment, but also by other bacterial taxa colonizing soil ecosystems.

All genes recovered in this study mediate high-level resistance to non-pathogenic *E. coli*, indicating that this might also be the case with respect to clinically relevant *Enterobacteriaceae*. Moreover, some of the detected soil DNA fragments encoding antibiotic resistance are potentially also involved in HGT. This demonstrates potential risks of soil resistomes and underlines why these comprehensive ARG reservoirs should be further explored in future studies. Our survey highlights that functional metagenomics can contribute to comprehend such studies as it represents a powerful approach with respect to the identification of previously undiscovered ARGs.

## 3. Materials and Methods

### 3.1. Soil Metagenomic Libraries

The metagenomic libraries AEW1, AEW5, SEG8 and SEW5, used for function-based screening, were reported previously [26,27], whereas library AEW4 was constructed in this study as described by Nacke et al. [26]. Names of the metagenomic libraries refer to the designation of soil samples from which the libraries were derived. The samples AEW1, AEW4 and AEW5 represent spruce (AEW1) and beech (AEW4 and AEW5) forest soil collected within the German Biodiversity Exploratory Schwäbische Alb, whereas the samples SEG8 and SEW5 represent grassland (non-fertilized pasture) and beech forest soil, respectively, collected within the German Biodiversity Exploratory Schorfheide-Chorin [26,27,53]. All samples were derived from A horizons (topsoil) and metagenomic libraries were constructed using the plasmid pCR-XL-TOPO (Thermo Fisher Scientific, Braunschweig, Germany). This vector allows cloning of up to approximately 10 kb DNA fragments.

### 3.2. Function-Based Metagenomic Library Screening

Metagenomic library-bearing *E. coli* DH5α clones were plated on agar medium containing 50 mg/L kanamycin (Carl Roth, Karlsruhe, Germany), which selects for the vector pCR-XL-TOPO (Thermo Fisher Scientific), and one of the five different antibiotics. These antibiotics comprised ampicillin (Gerbu, Heidelberg, Germany) (100 mg/L), ceftazidime (Sigma-Aldrich, Steinheim, Germany) (1 mg/L), chloramphenicol (Sigma-Aldrich) (12.5 mg/L), fosfomycin (Sigma-Aldrich) (128 mg/L) and trimethoprim (Sigma-Aldrich) (8 mg/L). In case of the latter antibiotic, Mueller–Hinton agar (Sigma-Aldrich) was used, as it is low in inhibitors that affect trimethoprim susceptibility, and with respect to the other antibiotics LB agar (10 g/L NaCl (Carl Roth), 10 g/L tryptone (Oxoid, Wesel, Germany), 5 g/L yeast extract (Oxoid) and 15 g/L agar-agar, Kobe I (Carl Roth)) was used. Colonies formed after incubation for 1–3 days at 37 °C under aerobic conditions were picked for further study.

### 3.3. Sequence Analysis

Inserts of recombinant plasmids, extracted from positive clones using the QIAprep Spin Miniprep kit (Qiagen, Hilden, Germany) according to the instructions of the manufacturer, were sequenced by Microsynth Seqlab (Göttingen, Germany) (Sanger sequencing technology). We used the Kaiju program [54] to predict the taxonomic origin of the inserts. For an initial prediction of potential ORFs located on the inserts, the ORF finder tool, provided by the National Center for Biotechnology Information (NCBI), was used. In a next step, BLAST [55] searches against the NCBI non-redundant protein sequence database were performed. Additionally, we performed BLAST searches against the Beta-Lactamase DataBase [56] (version Feb 13, 2020). The prediction of signal peptides with respect to deduced gene products was performed by using SignalP 5.0 [57]. BLAST searches against the NCBI non-redundant protein sequence database and the ACLAME database [58] version 0.4 were performed to identify mobile genetic elements.

Neighbor-joining phylogenetic trees were constructed using MEGA X [59] based on ClustalW [60] alignments. These alignments included sequences representing beta-lactamases, methionine sulfoxide reductases or DHFRs and pteridine reductases. Bootstrap values were calculated based on 1000 replications. We used the number of differences method to compute evolutionary distances.

### 3.4. Subcloning of Putative ARGs

Putative resistance genes and flanking sequences, potentially comprising promoters, were amplified via PCR. The PCR reaction mixture (50 µL) contained 10 µL 5-fold Phusion GC buffer (Thermo Fisher Scientific), 200 µM of each of the four deoxynucleoside triphosphates, 3% DMSO, 1 µL of 50 mM MgCl_2_ solution, 0.2 µM of each primer, 1 U of Phusion High-Fidelity DNA polymerase (Thermo Fisher Scientific), and approximately 20 ng of plasmid DNA. PCR primers are listed in Table 5. The following PCR cycling conditions were used: initial denaturation at 98 °C for 1 min, 15 cycles of denaturation at 98 °C for 45 s, annealing for 45 s (annealing temperatures, see Table 5), and extension at 72 °C for 30 s per kb, followed by a final extension period at 72 °C for 5 min. After PCR-based amplification, the products were purified using the QIAquick PCR purification kit (Qiagen) as described by the manufacturer.

With respect to PCR products including an ORF potentially encoding ceftazidime, chloramphenicol, fosfomycin or trimethoprim resistance, a deoxyadenosine was added to the 3′ termini of the DNA as described by Nacke et al. [26]. Subsequently, the modified PCR products were purified by using the QIAquick PCR purification kit (Qiagen) and inserted into vector pCR4-TOPO (Thermo Fisher Scientific) according to the instructions of the manufacturer. The resulting vectors were then transformed into chemically competent *E. coli* TOP10 cells (Thermo Fisher Scientific) as described by the manufacturer.

PCR products including an ORF potentially encoding ampicillin resistance were cloned into vector pHSG398 (Takara Bio Europe S.A.S., Saint-Germain-en-Laye, France) instead of pCR4-TOPO (Thermo Fisher Scientific). This is advantageous with respect to the identification of target subclones because in contrast to pCR4-TOPO (Thermo Fisher Scientific), pHSG398 (Takara) encodes no ampicillin resistance. The In-Fusion HD Cloning kit (Takara) was used to insert PCR products into pHSG398 (Takara) according to the instructions of the manufacturer. This required the presence of overhangs at the ends of PCR products, homologous to those of linearized pHSG398 (Takara) vector. The overhangs were added by using appropriate primers (see Table 5) within the PCR reaction described above. After insertion of PCR products into pHSG398 (Takara), the resulting vectors were transformed into *E. coli* Stellar competent cells (Takara) according to the protocol of the manufacturer.

Before further analysis, the inserts of obtained subclones have been sequenced by Microsynth Seqlab (Sanger sequencing technology). Based on the obtained sequencing data, we checked the reading frame and verified if the sequences of the cloned DNA fragments match exactly the expected insert sequences.

### 3.5. Determination of Minimum Inhibitory Concentrations (MICs)

The 2-fold serial microtiter broth dilution method was used to determine MICs as described by Willms et al. [27]. All MIC assays were conducted in Mueller Hinton Broth. *E. coli* TOP10 and *E. coli* Stellar carrying vector pCR4-TOPO (Thermo Fisher Scientific) and pHSG398 (Takara), respectively, were used as control. The MICs were recorded after 20 h of incubation at 37 °C and all assays were performed in duplicate.

### 3.6. Accession Numbers

Insert sequences of plasmids derived from metagenomic library clones, showing resistance toward target antibiotics, have been deposited in GenBank under accession numbers MW601939 to MW601946.

## Figures and Tables

**Table 1:** Characteristics of soil metagenomic libraries and designation of plasmids harbored by positive clones.

Library	Number of Clones	Average Insert Size (kb)	Insert Frequency (%)	Estimated Library Size (Gb)	Plasmids of Positive Clones
AEW4	54320	3.4	80	0.15	pLAEW4_amp01
SEG8 *	559000	4.8	86	2.30	pLSEG8_amp01, pLSEG8_cef01, pLSEG8_tri01-02
SEW5 *	166040	4.0	95	0.63	pLSEW5_chl01
AEW1 *	129748	6.7	91	0.79	pLAEW1_fos01
AEW5 *	90300	5.2	89	0.42	pLAEW5_tri01

* Previously generated metagenomic libraries [26,27].

**Table 2:** Proteins encoded by genes associated with antibiotic resistance and their observed sequence identities.

Gene	No. of Encoded Amino Acids	Closest Similar Protein, Accession no. (no. of Encoded Amino Acids), Organism	*E*-Value	Identity to Closest Similar Protein (Blast), no. of aa Similar/Total no. (%)	Percent Identity to Closest Similar Protein (Clustal, Full Length)
*AEW4_amp01*	343	Efflux RND transporter periplasmic subunit, PYM22253 (469), *Candidatus* Rokubacteria bacterium	1e-14	102/359 (28%)	20.833
*SEG8_amp01*	220	Bifunctional methionine sulfoxide reductase, MBI1189218 (321), *Tepidisphaera* sp.	4e-112	158/216 (73%)	48.160
*SEG8_cef01*	251	NDM-CcrA beta lactamase, AIA10512 (261), uncultured bacterium	9e-119	162/248 (65%)	62.069
*SEW5_chl01*	402	Bcr/CflA subfamily drug resistance transporter, AIA10695 (410), uncultured bacterium	0.0	370/402 (92%)	90.244
*AEW1_fos01*	443	UDP-N-acetylglucosamine 1-carboxyvinyltransferase (MurA), HAL80920 (434), *Mucilaginibacter* sp.	0.0	406/433 (94%)	91.648
*AEW5_tri01*	263	Pteridine reductase, PYT50939 (263), *Acidobacteria* bacterium	2e-171	236/263 (90%)	89.734
*SEG8_tri01*	165	Dihydrofolate reductase, WP_091310122 (162), *Flavobacterium terrigena*	3e-108	147/162 (91%)	89.091
*SEG8_tri02*	166	Dihydrofolate reductase, QOI95992 (167), *Flammeovirgaceae* bacterium	4e-88	117/165 (71%)	70.060

**Table 3:** Antibiotic susceptibility of *E. coli* harboring vector pCR4-TOPO without or with insert.

Plasmid	Minimum Inhibitory Concentration (µg/mL)							
	CHL	FOS	RIF	TET	TRI	CPH	CFX	CFT
Cloning vector	1	4	4	≤0.25	≤0.25	4	2	≤0.125
pCR4_SEG8_cef01	1	≤1	4	≤0.25	≤0.25	16	8	64
pCR4_SEW5_chl01	64	4	4	0.5	≤0.25	4	2	≤0.125
pCR4_AEW1_fos01	1	≥512	4	≤0.25	≤0.25	4	2	≤0.125
pCR4_AEW5_tri01	1	4	4	≤0.25	≥128	4	2	≤0.125
pCR4_SEG8_tri01	1	4	4	≤0.25	≥128	4	2	≤0.125
pCR4_SEG8_tri02	1	4	4	≤0.25	≥128	4	2	≤0.125

CHL, chloramphenicol; FOS, fosfomycin; RIF, rifampicin; TET, tetracycline; TRI, trimethoprim; CPH, cephalexin; CFX, cefoxitin; CFT, ceftazidime.

**Table 4:** Antibiotic susceptibility of *E. coli* harboring vector pHSG398 without or with insert.

Plasmid	Minimum Inhibitory Concentration (µg/mL)					
	AMP	CRB	PIP	CPH	CFX	CFT
Cloning vector (pHSG398)	≤2	≤2	≤2	4	2	≤0.125
pHSG398_AEW4_amp01	≥1024	≥1024	512	4	2	≤0.125
pHSG398_SEG8_amp01	≥1024	≥1024	512	4	2	≤0.125

AMP, ampicillin; CRB, carbenicillin; PIP, piperacillin; CPH, cephalexin; CFX, cefoxitin; CFT, ceftazidime.

**Table 5:** Primer sets designed in this study and corresponding templates.

Template	Oligonucleotide	Sequence (5′ to 3′)	Annealing Temperature ( °C)
pLAEW4_amp01	AEW4_amp01_for_150	CCATGATTACGAATTCTTATTTGTTCGGCGTCTTGC	60
	AEW4_amp01_rev	TACCGAGCTCGAATTCTAATGCACTGACTTCTGCAAG	60
pLSEG8_amp01	SEG8_amp01_for_46	CCATGATTACGAATTGTTCGACAAGGACCACATC	60
	SEG8_amp01_rev	TACCGAGCTCGAATTTCACTCGTCCTCCGGCCC	60
pLSEG8_cef01	SEG8_cef01_for_150	GAGATTGCTTCGTCGCTCTG	59
	SEG8_cef01_rev	TCACTTCCTCTGGGCAACATTC	59
pLSEW5_chl01	SEW5_chl01_for_150	ACCATTTGCTATAATAGCATCTAC	53
	SEW5_chl01_rev	TCATCTCTTTCTCGGTATCAG	53
pLAEW1_fos01	AEW1_fos01_for_150	CCAAAATAACAGGGGGCG	55
	AEW1_fos01_rev	TCACACCCTCGTAATATCCG	55
pLAEW5_tri01	AEW5_tri01_for_150	GCGGCGATTGCGAGTAG	59
	AEW5_tri01_rev	TCACAACGTCCGCCCG	59
pLSEG8_tri01	SEG8_tri01_for_150	ACTTACTCAGAATTTGACATA	49
	SEG8_tri01_rev	TTAATTGGCTCTCACATAAG	49
pLSEG8_tri02	SEG8_tri02_for_145	CGGACCCCTCAGAAC	52
	SEG8_tri02_rev	TTACTTCTTTTCATAGATAACAAAGTC	52

Primer overhangs required for In-Fusion cloning are underlined.

## Data Availability

The datasets generated and analyzed during the current study are publicly available in GenBank; accession numbers: MW601939 to MW601946.

## Supplementary Materials

The following are available online at https://www.mdpi.com/article/10.3390/antibiotics10040378/s1, Table S1: Taxonomic classification of plasmid inserts from positive clones, Table S2: Open reading frames potentially involved in lateral gene transfer identified on plasmids from positive clones and description of corresponding gene products and their observed sequence identities, Figure S1. Neighbor-joining phylogenetic tree based on amino acid sequences of SEG8_Amp01 and methionine sulfoxide reductases.
