# Supplementary material for: Novel Soil-Derived Beta-Lactam, Chloramphenicol, Fosfomycin and Trimethoprim Resistance Genes Revealed by Functional Metagenomics
Source: Antibiotics (Basel). 2021 Apr 3;10(4):378. doi: 10.3390/antibiotics10040378 (PMC8066302; doi:10.3390/antibiotics10040378)
Supplement: Supplementary file 1 [file antibiotics-10-00378-s001.pdf]

Supplementary Materials

Novel Soil-Derived Beta-Lactam, Chloramphenicol, Fosfomycin and Trimethoprim Resistance Genes Revealed by Functional Metagenomics

Inka Marie Willms [1], Maja Grote [1], Melissa Kocatürk [1], Lukas Singhoff [1], Alina Andrea Kraft [1], Simon Henning Bolz [1] and Heiko Nacke [1,]*

[1] Department of Genomic and Applied Microbiology and Göttingen Genomics Laboratory, Institute of Microbiology and Genetics, Georg-August University of Göttingen, D-37077 Göttingen, Germany
* Correspondence: hnacke@gwdg.de; Tel.: +49-551-3933841

Table S1. Taxonomic classification of plasmid inserts from positive clones.

Plasmid	Taxonomic classification of insert
pLAEW4_amp01	Cellular organisms; Bacteria; Acidobacteria; unclassified Acidobacteria; Acidobacteria bacterium
pLSEG8_amp01	Cellular organisms; Bacteria; Proteobacteria; Alphaproteobacteria; Rhodospirillales; Rhodospirillaceae; unclassified Rhodospirillaceae; Rhodospirillaceae bacterium
pLSEG8_cef01	Cellular organisms; Bacteria; Actinobacteria; Acidimicrobiia; Acidimicrobiales; unclassified Acidimicrobiales
pLSEW5_chl01	Cellular organisms; Bacteria; environmental samples; uncultured bacterium
pLAEW1_fos01	Cellular organisms; Bacteria; Bacteroidetes; Sphingobacteriia; Sphingobacteriales; Sphingobacteriaceae; Mucilaginibacter
pLAEW5_tri01	Cellular organisms; Bacteria; unclassified Acidobacteria; Acidobacteria bacterium
pLSEG8_tri01	Cellular organisms; Bacteria; Bacteroidetes; Flavobacteriia; Flavobacteriales; Flavobacteriaceae; Flavobacterium; Flavobacterium terrigena
pLSEG8_tri02	Cellular organisms; Bacteria; environmental samples; uncultured bacterium

Table S2. Open reading frames potentially involved in lateral gene transfer identified on plasmids from positive clones and description of corresponding gene products and their observed sequence identities.

Plasmid	ORF#	No. of encoded amino acids	Closest similar protein potentially involved in lateral gene transfer, accession no. (no. of encoded amino acids), organism	E value	Percent identity to the closest similar protein
pLAEW4_amp01	9	43	HNH endonuclease, NYW97720 (32), *Escherichia coli*	0.002	9/11 (82%)
pLSEW5_chl01	13	25	Transposase, EYU69316 (38), *Streptomyces* sp. PCS3-D2	0.002	7/10 (70%)
pLAEW1_fos01	33	41	Transposase, HHX66676 (48), *Gallicola* sp.	5e-06	8/18 (44%)

Table S3. Observed sequence identities of AEW4_Amp01 to beta-lactamases.

Gene	No. of encoded amino acids	Beta-lactamase showing similarity, accession no. (no. of encoded amino acids), organism	E value	Percent identity to the closest similar protein
AEW4_amp01	343	GOB family subclass B3 metallo-beta-lactamase, OPC00210.1 (290), *Elizabethkingia ursingii*	4.12	21/77 (27.27%)
		Beta-lactamase OXA-114u, AGW27424.1 (275), *Achromobacter xylosoxidans*	8.33	15/39 (38.46%)

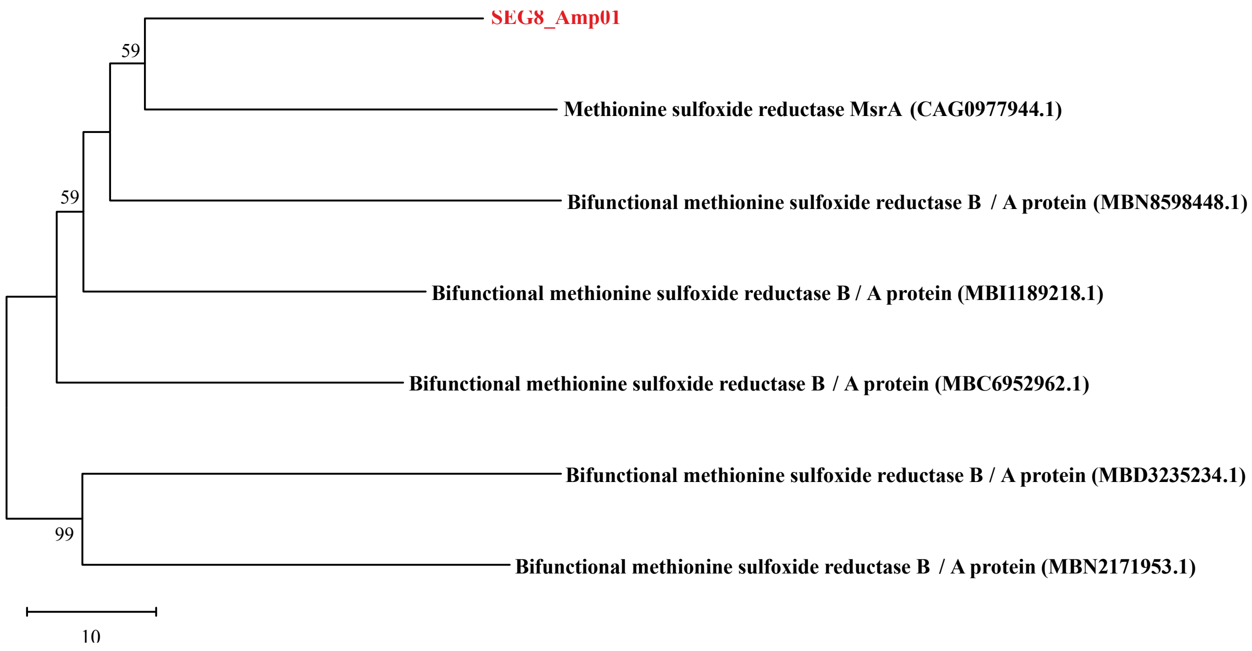

Figure S1. Neighbor-joining phylogenetic tree based on amino acid sequences of SEG8_Amp01 and methionine sulfoxide reductases. Bootstrap values ≥50, based on 1000 iterations, are shown at branching points. Accession numbers of the different methionine sulfoxide reductases are given in parentheses.
